# Digital technique to analyze the wear of screw-retained implant supported metal-ceramic dental prostheses and natural tooth as antagonist: a pilot study

**DOI:** 10.1186/s12903-024-03881-y

**Published:** 2024-02-03

**Authors:** Paulina Rodríguez Torres, Agustín Galparsoro Catalán, Elena Riad Deglow, Javier Flores Fraile, Jorge Alonso Pérez-Barquero, Ana Belén Lobo Galindo, Álvaro Zubizarreta-Macho, Sofía Hernández Montero

**Affiliations:** 1 Department of Implant Surgery, Faculty of Health Sciences, Alfonso X el Sabio University, 28691 Madrid, Spain; 2 Department of Surgery, Faculty of Medicine, University of Salamanca, 37008 Salamanca, Spain; 3 Department of Stomatology, Faculty of Medicine and Dentistry, University of Valencia, 46010 Valencia, Spain

**Keywords:** Implant supported, Morphometry, Digital, Wear, Prothesis, Screw-retained

## Abstract

The aim of this study was to describe a novel digital technique to analyze the wear of screw-retained implant-supported metal-ceramic dental prostheses and natural tooth as antagonist.

**Materials and methods**

Ten patients were consecutively included to rehabilitate partial edentulism by dental implants. Both the screw-retained implant-supported metal-ceramic dental prostheses and the natural tooth as antagonist were submitted to a digital impression through an intraoral scan to generate a Standard Tessellation Language digital file preoperatively (STL1), at 3 months (STL2), and 6 months (STL3) follow-up. Afterwards, an alignment procedure of the digital files (STL1-STL3) was performed on a reverse engineering morphometric software (3D Geomagic Capture Wrap) and volume changes at the screw-retained implant-supported metal-ceramic dental prostheses and the natural tooth as antagonist were analyzed using Student’s *t*-test. Moreover, Gage R&R statistical analysis was conducted to analyze the repeatability and reproducibility of the digital technique.

**Results**

Gage R&R showed a variability attributable to the digital technique of 3.8% (among the measures of each operator) and 4.5% (among operators) of the total variability; resulting repeatable and reproducible, since the variabilities were under 10%. In addition, statistically significant differences were shown at the wear volume (μm^3^) of both the natural tooth as antagonist (*p* < 0.0001) and the screw-retained implant-supported metal-ceramic dental prostheses between 3- and 6-months follow-up (*p* = 0.0002).

**Conclusion**

The novel digital measurement technique results repeatable and reproducible to analyze the wear of screw-retained implant-supported metal-ceramic dental prostheses and natural tooth as antagonist.

## Background

Regarding the data obtained during the year 2020, in Spain between 1200000 and 1400000 million dental implants are placed per year. Regarding the age group, two out of every ten people between 25 and 79 years old have a dental implant placed, there is also a clear consensus in saying that there will not be a decrease in the demand for most dental treatments, and there is a trend to think that all will increase the demand except for restorative treatments [1, 2].

As a result of the increase in the placement of dental implants, the prevalence studies of peri-implant disease have increased significantly [1], as well as the risk factors associated with dental implant failure related to different causes such as the surgical factor and the postsurgical factors, as well as with pre-existing risk factors such as diabetes and osteoporosis and with the habits of patients such as alcoholism, smoking, etc., [2].

However, we did not find enough publications of clinical studies in which the association between the placement of implant-supported prostheses and the wear of natural teeth in the face of these restorations is validated. The wear of the tooth structure is an inevitable natural process that occurs when the tooth and the tooth, or the tooth and the restoration are in contact and slide against each other. However, this natural process can be accelerated by introducing restorations whose wear properties differ from those of the tooth structure against which they are sliding. Enamel has been shown to be subject to accelerated wear when opposed to ceramic [3].

Ceramic hardness has always been associated with greater abrasiveness against teeth [4, 5], we find studies that also show that the degree of wear is more affected by the surface structure and roughness of the restoration or environmental factors such as porosity and friction resistance in restored teeth [1, 6].

It is desirable that the wear behavior of the restorative materials is similar to natural enamel, since excessive wear could lead to clinical problems such as damage to the occlusal surfaces of the teeth, decrease in vertical dimension, alteration of masticatory function associated with the remodeling of temporomandibular joints, hypersensitivity of the dentin or dental necrosis and almost always entails at least one aesthetic deterioration [7–9].

To observe and evaluate wear, it is necessary to understand the mechanisms of tooth wear and how it can be measured and evaluated, both clinically and in the laboratory. The terms abrasion, wear and even corrosion were often used generically encompassed in one, to identify what is dental wear caused mainly by food and parafunctional habits. In contrast, today there is an agreement that the terms, abrasion, wear and corrosion describe different mechanisms [10].

Determining a risk factor for a clinical procedure requires validation through a study that meets the criteria of randomized samples and statistical validation of the results. In this sense, it is necessary to clarify what are the factors associated with tooth wear in relation to implant-supported metal porcelain unit rehabilitation [2].

By providing data from this experimental study, according to the criteria analyzed, we will be able to have greater security and specific knowledge of the possible success and failure scenarios for the wear of natural teeth compared to implant-supported metal porcelain unit fixed prostheses, since, by generating a complete study of the models, better treatment decisions can be made for patients. When conducting searches on this topic, it can be observed that there is little literature on the subject, and it can be thought then that the disclosure of our findings will contribute to the state of the art because it will contribute new knowledge that is possibly highly appreciated by clinicians.

The aim of this study was to analyze and describe a novel digital technique to analyze the wear of screw-retained implant-supported metal-ceramic dental prostheses and natural tooth as antagonist, with a null hypothesis (H_0_) stating that neither the screw-retained implant-supported metal-ceramic dental prostheses nor the natural tooth as antagonist suffer a wear process.

## Methods

### Study design

A randomized controlled experimental trial was conducted at the Dental Centre of Innovation and Advanced Specialties at Alfonso X El Sabio University (Madrid, Spain) between January and September 2022, in accordance with the ethical guidelines established by the Declaration of Helsinki and the CONSORT Statement. The study was approved by Ethical Committee of the Faculty of Health Sciences, University Alfonso X el Sabio, in December 2021 (process no. 27/2021). Moreover, the study was approved by ClinicalTrials.org with identification number NCT05842655 (Registration date: 05/01/2023). All patients gave their informed consent to participate.

### Clinical procedure

Ten patients between 21–35 years of age and in good health, who presented partial edentulism in the posterior upper maxilla (molars), without systematic pathologies, without parafunctional habits (bruxism, clenching) and/or without temporomandibular joint disorders, without a natural tooth as antagonist or without an opposite tooth having caries or attrition which have follow-up standard tessellation language digital files available were consecutively included to rehabilitate partial edentulism by dental implants (BioHorizons; Birmingham, AL, USA) at the master’s degree of Oral Implantology and Implant-Supported Prostheses of Alfonso X El Sabio University (Madrid, Spain). Patients younger than 18 years old, presenting systemic pathologies, treated with other dental restorations, with parafunctional habits (bruxism, clenching) and/or temporomandibular joint disorders, without a natural tooth as antagonist or with an opposite tooth having caries or attrition or did not have follow-up standard tessellation language digital files available were excluded. ANOVA was used to establish the sample size, achieving 80% power with a confidence level of 5%, with an inter-group variability of 0.6 and intra-group variability of 4, to detect differences in contrasts of the null hypothesis H_0_: μ1 = μ2 = μ3 = μ4. The patients were scheduled at 3 and 6 months for follow-up appointments.

### Experimental procedure

**Fig. 1:**
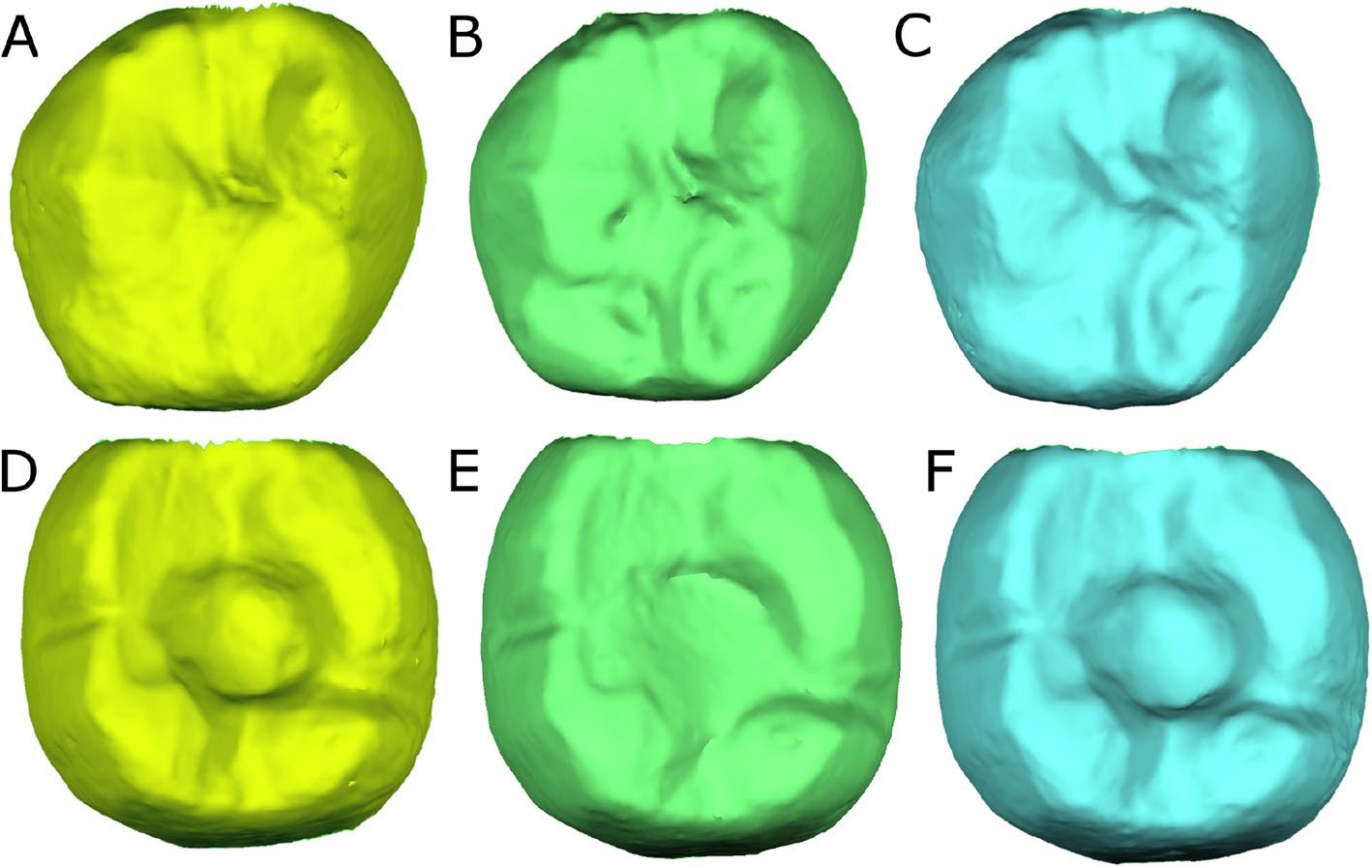
**A** Preoperative, **B** 3 months follow-up and (**C**) 6 months follow-up STL digital files of the segmented natural tooth. (**D**) Preoperative, (**E**) 3 months follow-up and (**F**) 6 months follow-up STL digital files of the screw-retained implant-supported metal-ceramic dental prostheses

Both the screw-retained implant-supported metal-ceramic dental prostheses with feldspathic porcelain veneer and the natural tooth as antagonist were submitted to a full-arch digital impression through an intraoral scan (True Definition, 3M ESPE ™, Saint Paul, MN, USA) (STL1) via a 3D in-motion video imaging technology, after occlusal adjustment following Kim et al recommendations [11], to generate an standard tessellation language (STL) (STL1) digital file using a cloud of points that create a tessella network, representing 3-dimensional objects as polygons composed of equilateral triangle tessellas [12, 13]. Occlusal considerations included light contacts at heavy bite and no contact at light bite in maximum intercuspation [14], reduced inclination of cusps, centrally oriented contacts with a 1–1.5mm flat area, and a narrowed occlusal table [15, 16], centrally oriented occlusal contacts to reduce bending moments attributable to mechanical problems and implant fractures [17] and increased proximal contacts to provide additional stability of restorations [18]. The capturing images procedure was performed following manufacture recommendations by scanning prior the palatine and occlusal surface and following the buccal surface. The patients were scheduled for postoperative digital impression through an intraoral scan (True Definition, 3M ESPE ™, Saint Paul, MN, USA) at 3 months (STL2) and 6 months (STL3) follow-up appointments (Fig. 1).

### Alignment procedure

**Fig. 2:**
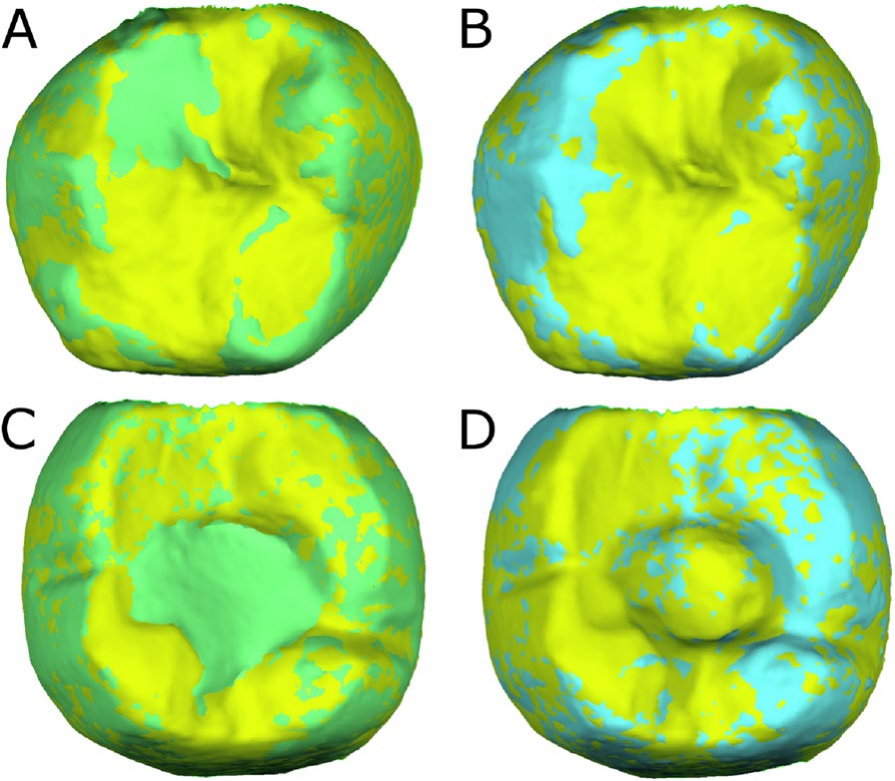
**A** Alignment procedure between STL1 and STL2 and (**B**) STL1 and STL3 digital files of the segmented natural tooth. (**C**) Alignment procedure between STL1 and STL2 and (**D**) STL1 and STL3 digital files of the screw-retained implant-supported metal-ceramic dental prostheses

Once STL1–3 of both the screw-retained implant-supported metal-ceramic dental prostheses and the natural tooth as antagonist were imported to reverse engineering geomorphometric software (3D Geomagic Capture Wrap, 3D Systems©, Rock Hill, SC, USA); a full-arch alignment procedure was conducted. STL1 of both the screw-retained implant-supported metal-ceramic dental prostheses and the natural tooth as antagonist were considered the reference digital files and STL2–3 digital files were superimposed on it, with the best fit algorithm. Afterward, the STL1 digital files was segmented and individually three-dimensionally compared with the STL2 digital file, STL3 digital file, and STL4 digital file with STL1 used as the reference with the spectrum set to ±100 μm and the tolerance to ±10 μm (Fig. 2).

### Measurement procedure

**Fig. 3:**
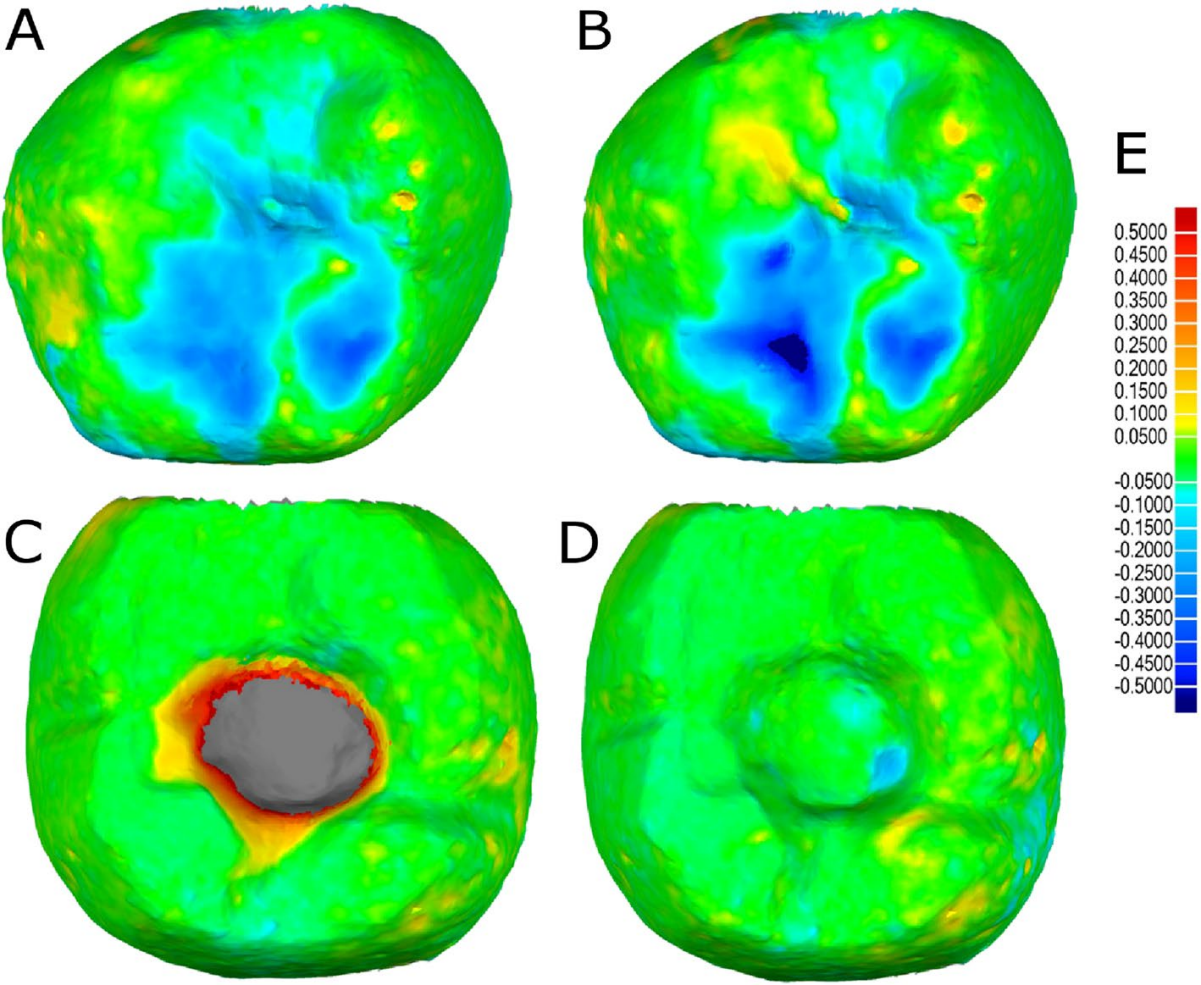
(**A**) Alignment procedure between STL1 and STL2 and (**B**) STL1 and STL3 digital files of the segmented natural tooth. (**C**) Alignment procedure between STL1 and STL2 and (**D**) STL1 and STL3 digital files of the screw-retained implant-supported metal-ceramic dental prostheses. (**E**) Spectrum values used in (**A–D**). Warm colors represent a volume increase, cold colors represent a volume decrease, and green represents an accurate alignment

After the alignment procedures, wear volume (μm^3^) of both the screw-retained implant-supported metal-ceramic dental prostheses and the natural tooth as antagonist were measured at 3 months and 6 months follow-up appointments (Fig. 3).

### Validation of the repeatability and reproducibility

To validate the repeatability of this new protocol, the measurements described above were calculated six times using the same operator (Operator A). The measurements were calculated six times by another operator (Operator B) to validate the reproducibility of this new measurement technique.

### Statistical tests

Statistical analysis of the measurement variables was conducted using SAS 9.4 (SAS Institute Inc., Cary, NC, USA). Descriptive statistics are expressed as mean and SD for the volume changes (μm^3^) of both the screw-retained implant-supported metal-ceramic dental prostheses and the natural tooth as antagonist were measured at 3- and 6-months follow-up appointments. Comparative analysis between the wear volume (μm^3^) of both the natural tooth as antagonist and the screw-retained implant-supported metal-ceramic dental prostheses between 3- and 6-months follow-up were analyzed using Student-*t* test. Gage R&R statistical analysis was conducted to analyze the repeatability and reproducibility of this digital measurement technique.

## Results

**Fig. 4:**
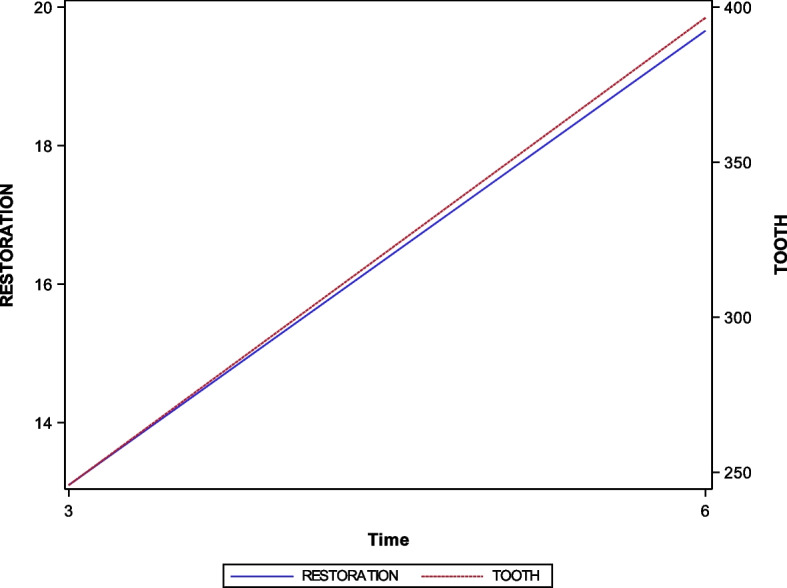
Evolution of the measures of the wear volume (μm^3^) of both the natural tooth as antagonist and the screw-retained implant-supported metal-ceramic dental prostheses at 3- and 6-months follow-up appointments

The means and SD values for the wear volume (μm^3^) of both the screw-retained implant-supported metal-ceramic dental prostheses and the natural tooth as antagonist are displayed in Table 1 and Fig. 4.

**Table 1:** Descriptive statistics of the wear volume (μm^3^) of both the screw-retained implant-supported metal-ceramic dental prostheses and the natural tooth as antagonist

Moment	Location	*n*	Mean	SD	Minimum	Maximum
3 months	Restoration	10	13.10	7.86	3.90	27.50
	Tooth	10	245.86	72.24	105.20	331.40
6 months	Restoration	10	19.66	7.97	8.20	33.40
	Tooth	10	396.59	90.76	213.90	514.60

*SD* standard deviation

Statistically significant differences were shown at the wear volume (μm^3^) of both the natural tooth as antagonist (*p* < 0.0001) and the screw-retained implant-supported metal-ceramic dental prostheses between 3- and 6-months follow-up (*p* = 0.0002) (Fig. 4).

**Fig. 5:**
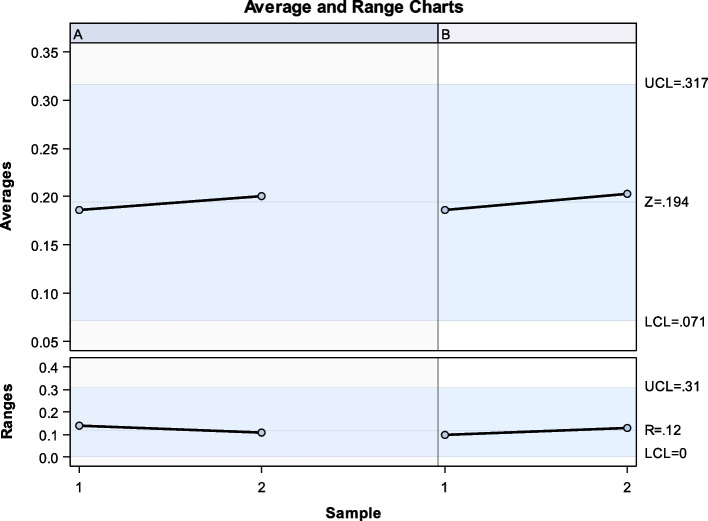
Charts for the average of the measures of the wear volume (μm^3^) of both the natural tooth as antagonist and the screw-retained implant-supported metal-ceramic dental prostheses at 3- and 6-months follow-up appointments

**Fig. 6:**
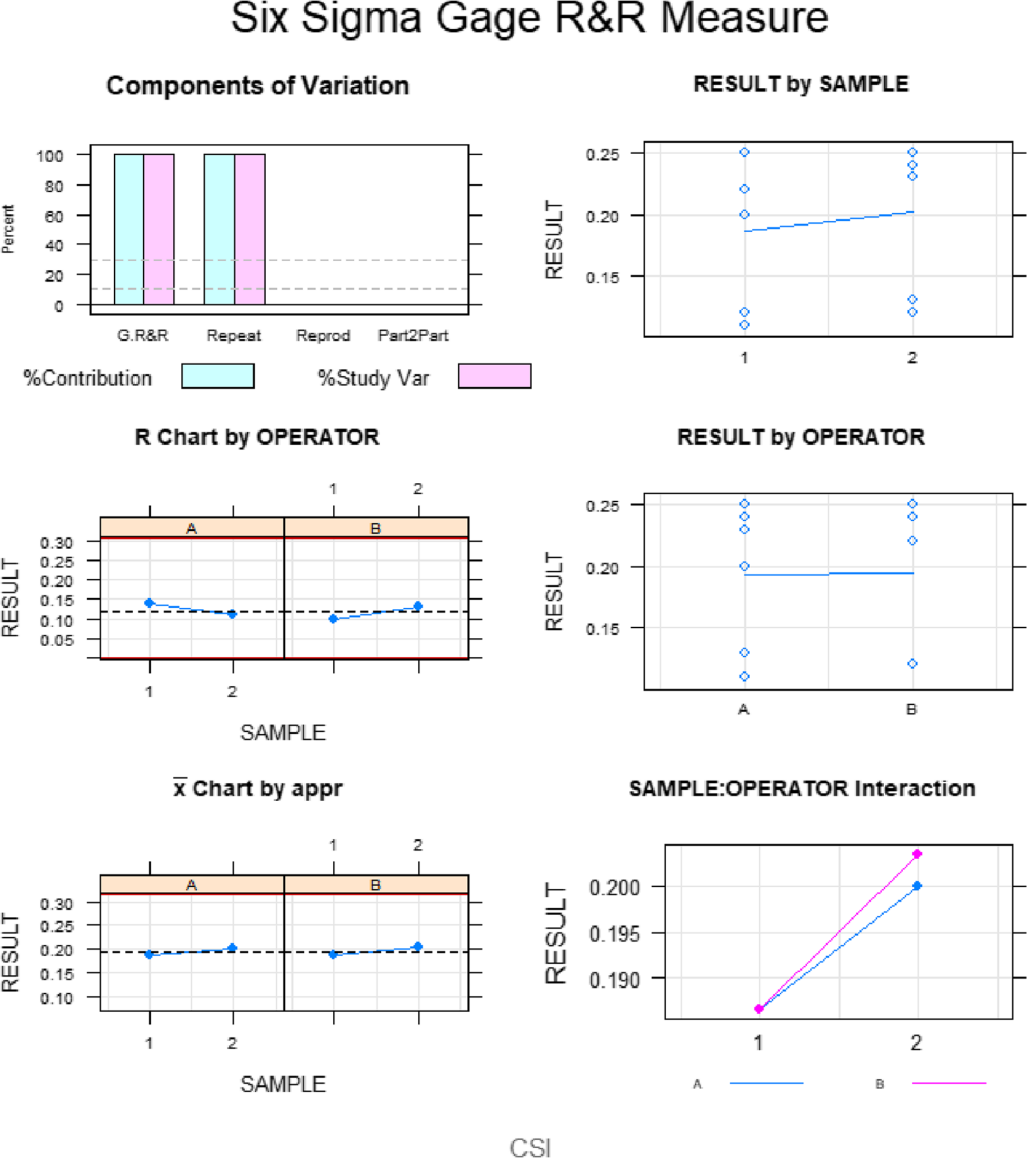
Measurement system analysis related to the wear volume (μm^3^) of both the natural tooth as antagonist and the screw-retained implant-supported metal-ceramic dental prostheses at 3- and 6-months follow-up appointments. With a chart of the contribution of each component to the total variance (Components of Variation), a mean control chart and a range control chart (R Chart by Operator and x Chart by appr), every measurement point in the graph (Trial by I and Trial by Operator II), and the interactions between the operators (**i**): Operator interaction)

The Gage R&R statistical analysis of the digital measurement technique in terms of the wear volume (μm^3^) of both the natural tooth as antagonist and the screw-retained implant-supported metal-ceramic dental prostheses at 3- and 6-months follow-up appointments showed that the variabilities attributable to the digital measurement technique were 3.8% (among the measures of each operator) and 4.5% (among the measures of the operators); respectively, of the total variability of the samples. The digital measurement technique to quantify the wear volume (μm^3^) of both the natural tooth as antagonist and the screw-retained implant-supported metal-ceramic dental prostheses at 3- and 6-months follow-up appointments was considered repeatable and reproducible because the variabilities were under 10%, which is considered repeatable and reproducible (Figs. 5 and 6).

## Discussion

The results obtained in the present study reject the null hypothesis (H_0_) that states that neither the screw-retained implant-supported metal-ceramic dental prostheses nor the natural tooth as antagonist suffer a wear process.

The results derived from the present study showed that the natural tooth as antagonist of screw-retained implant-supported metal-ceramic dental prostheses suffers a progressive wear process over time, despite performing an occlusal adjustment following the recommendations of Kim et al, who reported that dental implants may be more prone to occlusal overloading, which is often considered one of the potential causes of peri-implant bone loss and implant/implant prosthetic failure. In addition, loading factors that can negatively influence implant longevity include long extension pieces, parafunctions, inadequate occlusal designs, and premature contacts. Therefore, it is important to control implant occlusion within physiological limits and thereby provide optimal implant loading to ensure long-term implant success [11]. Fortunately, this novel digital measurement technique provides a useful method to analyze and quantify this undesirable event, capable of being used to other dental materials and restorations.

The wear effect of restoring materials after function has been previously measured by means of articulating paper, material for the record of bite and clinical photographs taken with a digital intraoral camera. Etman et al used occlusal contact points, measuring up to 4 points on the occlusal surface of each crown and its opposing tooth enamel surface. Landmarks were selected on noncontact tooth surface areas that were most likely to have stability over the course of the study, for example the occlusal fossa [19]. Previously, different measurement techniques have been proposed to analyze the wear ranges of different ceramic restoring materials that have natural teeth as antagonists. Etman et al used analogic dental impressions to quantify the wear of natural and ceramic surfaces and concluded that the enamel of the opposing teeth was worn away in all areas of contact with the ceramics. These materials also caused reciprocal enamel wear in the occlusal contact areas, appearing in the contact areas of all the teeth, a circular defect of approximately 1-2 mm diameter in the occlusal contact areas [14]. However, Yilmaz et al evaluated the mechanical properties of ceramic-based materials such as biaxial flexural strength and fracture strength tests, resulting significant differences in the strength and hardness values of the materials evaluated [20]. In addition, a previous in vitro study has been conducted to evaluate the fracture resistance of different restoring materials according to the ISO 6872:2015 statements [21]. The physical properties of ceramic materials vary greatly depending on their bonds, but in general they are distinguished by their hardness and brittleness, as well as having high melting points. They are rigid after firing, although before it they are extremely ductile and can take infinite shapes and sizes. These properties coincide with those of dental enamel, and current research seeks to match the characteristics and physical properties of enamel and ceramic.

In addition, this digital measurement technique has been previously validated to isolate and quantify the wear of M-Wire NiTi alloy endodontic reciprocating files after root canal treatment in terms of area (0.00%) and volume (0.00%) [22] Moreover, the digital measurement technique was also used to measure the wear of dental implant drilling burs after osteotomy site preparation for dental implant placement [23]. Briefly, the digital measurement technique was considered accurate, repeatable, and reproducible to measure the wear of both M-Wire NiTi alloy endodontic reciprocating files and dental implant drilling burs after osteotomy site preparation, since the variabilities attributable to the digital measurement technique were under 10%, which is considered repeatable and reproducible. In addition, the digital measurement technique proposed in the present study to analyze the volume of wear (μm^3^) both of the natural tooth as antagonist and of the screw-retained implant-supported metal-ceramic dental prostheses at the follow-up appointments at 3 and 6 months showed that the variabilities attributable to the digital measurement technique were 3.8% (between the measurements of each operator) and 4.5% (between the measurements of the operators); respectively, of the total variability of the samples, which was considered repeatable and reproducible because the variabilities were below 10%, which is considered repeatable and reproducible. Moreover, the follow-up period stablished in the present study has been previously used by Heintze et al to analyze the wear of dental protheses in the first 6 months after placement [24]. However, Stober et al did not find statistically significant differences in the wear of dental protheses after 6 and 12 months [25].

Additionally, the use of Gage R&R statistical analysis to validate the accurate, repeatability, and reproducibility of novel measurement techniques has been also used in previous studies. Requena Pérez et al used this statistical test to validate a digital measurement method to quantify the volume of the midpalatal suture after rapid maxillary expansion [26], González-Menéndez used this statistical analysis for analyzing the volumes of the left and right maxillary sinuses and the nasal and maxillary sinus airway complex after a sinus lift procedure using the lateral window approach [27], Faus-Matoses used also the Gage R&R statistical test to quantify the wear volume of controlled memory wire NiTi alloy endodontic reciprocating files after clinical use [17], Tzironi et al used this statistical análisis for analyzing the volume of maxillary and nasal sinus airways following suture palatine expansion performed with the Hyrax disyuntor appliance [28] and Zubizarreta-Macho et al used this statistical test to measure the area and volume of the remaining cement after removal of fixed multibracket appliances, the area and volume of remaining cement after cement removal, the area and volume of enamel removed after cement removal, and the volume of cement used to adhere the fixed multibracket appliance [29].

Moreover, Kazuhisa and Buckley reported the relationship between the friction exhibited during occlusal function and the ceramic wear. This friction occurs similarly when a silicon carbide surface is brought into contact with a diamond under conditions of relatively low contact pressure, elastic deformation can occur in both the silicon carbide and the diamond [30]. However, a large increase in the applied contact pressure results in a complete reversal of the friction characteristics. The increase in pressure causes a plastic deformation in the silicon carbide and produces permanent grooves during friction, which can lead to the appearance of very small cracks. When a much higher contact pressure is produced due to the higher concentration of stress in the contact area, the action of friction produces gross cracks on the surface and below it, in addition to plastic deformation [25].

To date, most of the articles analyze the wear of dental materials and tooth surface using subjective linear measurements; however, the suggested digital measurement procedure provides an objective, accurate, repeatable, and reproducible protocol for the analysis of the wear of screw-retained implant-supported metal-ceramic dental prostheses and natural tooth as antagonist. However, this novel digital measurement technique requires a digital impression through an intraoral scan and the resolution of this electronic devices, and the accuracy of the alignment procedure could influence the results of the measurement; however, the digital measurement procedure stablished a spectrum of ±100 μm and the tolerance of ±10 μm.

## Conclusion

The conclusion derived from the present study is that the novel digital measurement technique results repeatable and reproducible to analyze the wear of screw-retained implant-supported metal-ceramic dental prostheses and natural tooth as antagonist.

## Data Availability

The datasets used and/or analyzed during the current study are available from the corresponding author on reasonable request.
